# Hydrosoluble and Liposoluble Vitamins: New Perspectives through ADMET Analysis

**DOI:** 10.3390/medicina57111204

**Published:** 2021-11-04

**Authors:** Mirela Nicolov, Mioara Cocora, Valentina Buda, Corina Danciu, Adina Octavia Duse, Claudia Watz, Florin Borcan

**Affiliations:** 1 Departament of Pharmaceutical Physics, Faculty of Pharmacy, Victor Babeș University of Medicine and Pharmacy, 2nd Eftimie Murgu Square, 300041 Timișoara, Romania; nicolovmirela@gmail.com; 2 Department of Cardiac Surgery, Institute of Cardiovascular Diseases Timișoara, 13A Gh Adam Street, 300310 Timișoara, Romania; mcocora@cardiologie.ro; 3 Department of Clinical Pharmacy, Communication in Pharmacy and Pharmaceutical Care, Faculty of Pharmacy, Victor Babeş University of Medicine and Pharmacy, 2nd Eftimie Murgu Square, 300041 Timișoara, Romania; buda.valentina.oana@gmail.com; 4 Research Centre for Pharmaco-Toxicological Evaluation, Victor Babeş University of Medicine and Pharmacy, 2nd Eftimie Murgu Square, 300041 Timisoara, Romania; 5 Department of Pharmacognosy, Faculty of Pharmacy, Victor Babeș University of Medicine and Pharmacy, 2nd Eftimie Murgu Square, 300041 Timișoara, Romania; corina.danciu@umft.ro; 6 Department of Balneology, Medical Recovery and Rheumatology, Faculty of Medicine, Victor Babeș University of Medicine and Pharmacy, 2nd Eftimie Murgu Square, 300041 Timișoara, Romania; duse.adina@gmail.com; 7 Department of Analytical Chemistry, Faculty of Pharmacy, Victor Babeș University of Medicine and Pharmacy, 2nd Eftimie Murgu Square, 300041 Timișoara, Romania; fborcan@umft.ro

**Keywords:** water soluble vitamins, fat soluble vitamins, ADMET profile, bioavailability, metabolic stability, toxicity

## Abstract

*Background and Objectives*: The present study demonstrates that apart from the well-known toxicity of liposoluble vitamins, some hydrosoluble vitamins may also exert toxicity; thus, routine supplementation with vitamins or ingestion of fortified foods should not be considered harmless. The study addresses the possible correlations between the physico-chemical properties and the side effects of vitamins when taken in high doses or for a too long a period. *Materials and Methods*: The FAFDrugs4.0 computational tool was used for computational assessment of the ADMET profile of several hydro- and liposoluble vitamins. *Results*: ADMET analysis revealed the following major data: vitamin B3 and B13 showed reduced structural complexity; thus, a relative toxicological potential may be exerted. Vitamins B1 and B7 were found to have good oral absorption and thus good bioavailability, while Vitamin B3 was found to have decreased oral absorption. In addition, all of the liposoluble vitamins reflected higher complexity, much greater than most of the potentially therapeutically-proven compounds. *Conclusions*: The present study emphasizes the importance between the physico-chemical properties of vitamins and their possible toxicological impact.

## 1. Introduction

Vitamins are organic substances, essential nutrients usually taken from the diet, with a vital role in the proper functioning of the human body. They are used by the human organism for several important processes, such as the synthesis of other biochemicals (enzymes, hormones, and neurotransmitters), metabolism, the energetic pathway, antioxidant effects, and more [1,2]. Since their discovery at the beginning of the 20th century, only a few studies have been done regarding their action and toxicity in humans. The most common classification distinguishes hydro- (i.e., water) soluble vitamins (vitamins B and C) and lipo- (i.e., fat) soluble vitamins (vitamins A, D, E and K) [2]. A well-varied diet can provide the needed amount of vitamins for the good functioning of the human body (especially a raw diet), although the needs can be higher in the case of infants, adolescents, and pregnant and lactating women. The lack of vitamins (either because of poor diet, malabsorption or due to higher needs) can induce several pathological states such as pellagra (lack of niacin), beriberi (lack of thiamine) or scurvy (lack of vitamin C) [2].

To supply higher needs, standardized pharmaceutical formulations have been conceived and made available as over counter drugs or supplements.

Although vitamins are generally considered safe (especially the hydrosoluble ones), taken at a higher dose or for longer periods of time, vitamin consumption may not be harmless. The risk of overdose is increased for dietary supplements, especially in the case of liposoluble vitamins, which have a lower rate of elimination and a higher predisposition for accumulation in the body [1,2].

The 2D formulas and the main biological functions for all hydro- and lipo-soluble vitamins analyzed in the current study are presented in Table 1.

Therefore, an up-to-date analysis of the pharmacokinetic profile of hydro- and liposoluble vitamins with respect to their absorption, distribution, metabolism and excretion (ADME) features is essential in order to characterize their pathway within the body [15].

The ADMET profile is usually used for a better appreciation of benefit/risk ratio and therapeutic index. Quantifying the possible toxicity of a drug is still the most challenging process in the pharmacokinetic characterization and drug discovery process and has unpredictable results, as such multiple variables as species, organs, dose requirement, co-morbidity, and genetics can occur [15].

There are several studies based on physico-chemical properties such as size, lipophilicity, ionization, hydrogen bonding, polarity, aromaticity, and shape influence, absorption, distribution, metabolism, excretion and toxicity (ADMET or ADME TOX) in drugs and research compounds [16,17,18,19,20,21].

ADMET is a concept inherited from the QSAR (Quantitative Structure-Activity Relationship) method. It may be applied for drug optimization (structural design), screening of compounds (drug discovery phase), or to evaluate drug–drug interactions and toxicity of various pharmacological products [22]. Thus, predicting the ADMET profile for a potentially therapeutic compound starts with its physico-chemical properties as descriptors and proceeds by a series of rules.

Prediction of ADMET profile is mainly based on models of the physicochemical properties of chemicals which influence much of their pharmacokinetics, but there are also prediction models of the endpoints in ADME that are based on both in vitro and in vivo assay results [23,24,25,26].

This computational prediction of the ADMET profile for a chemical compound is possible with the help of several computer applications. At present there are a plethora of free tools (https://www.vls3d.com) such as ADMETlab, SwissADME, ADVERpred, etc. [27]. However, in this study the most recent version of the FAF-Drugs for Free-ADMET-Filtering tool and the most recent version of FAFDrugs 4.0 (Paris Diderot University, France) [28] were employed by using a new service, FAF-QED, that implements quantitative estimates of drug-like methods [29].

The aim of this paper is represented by an updated theoretical study of vitamins together with their ADMET profiles. Taking into account that all the data concerning the possible side effects of vitamins have been obtained mostly from experiments on animal models and there are limited studies revealing these effects in humans, this study addresses the possible correlations between the physico-chemical properties and possible side effects of vitamins [2,22]. To the best of our knowledge, this approach has never been conducted before. Similar studies have been performed for xenobiotics such as food supplements, steroids, food additives, and pesticides, revealing valuable results [30,31,32].

Since the current study employed only the FAFDrugs 4.0 analysis, the present observations are based only on the results of this toolkit; however, they reveal that some of the investigated vitamins violate the rules on which generation of the “drug-like” profile is based.

## 2. Materials and Methods

Hydro- and liposoluble vitamins with their molecular weights, maximum daily doses, and known side effects were used as materials. All of this information, together with the structural data files, was extracted from the PubChem database.

The method that consisted of producing the ADMET profiles of the investigated vitamins was related to the absorption of a chemical compound in the gastrointestinal tract, its distribution in the body, the metabolism of the compound, excretion of metabolites, and toxicity [33]. These profiles are obtained using an online computational facility, FAFDrugs4.0; sdf files for each vitamin from https://pubchem.ncbi.nlm.nih.gov/compound were used, and the program FAFDrugs4.0 (Paris Diderot University, France) generated the radar plots used for comparing the most useful drugs. This platform is a free accessible online tool and has good accuracy. In addition, it is continuously updated and has a friendly interface.

The parameters used in the simulation were [34] MW, molecular weight, logP, the logarithm of the partition coefficient between n-octanol and water, which characterizes lipophilicity. The XLOGP3 and OpenBabel methods were compared on a set of 1300 molecules with experimental logP values from the US National Cancer Institute, which showed that XLOGP3 (r^2^ = 0.94) provides better prediction accuracy. Lipinski RO5 is on CLOGP values, but Mannhold et al. evaluated several models and showed that the XLOGP3 and CLOGP methods give similar results [35]. logD represents the logP of the compounds at physiological pH (7.4); logSw represents the logarithm of the solubility of compounds in water, calculated by the ESOL method [36]; tPSA (the topological surface area) represents the sum of the contributions of the polar surface of the polar surface (i.e., atoms with respect to the binding model); HBD (Hydrogen Bond Donors) represents the sum of all –OH and –NH– (according to Lipinski’s RO5 rule); HBA (Hydrogen Bond Acceptors) represents the sum of all O and N (according to Lipinski’s RO5 rule); HBonds (Hydrogen Bond Donors and Acceptors) represents the sum of hydrogen donors and hydrogen acceptors, that are the hydrogen bonds involved in the “respirator” filter, according to Ritchie et al. [37]). n_SystemRing represents the smallest set of smallest rings; (SSSR) represents the smallest block of rings required to form other systems (e.g., three for a compound involving three phenyls); MaxSizeSystemRing (the size of the largest ring system) represents the number of atoms involved in the largest ring system. The number of atoms involved in the largest ring in the system (e.g., twelve for two fusion rings of six ring members) is max_ring; RotatableBonds (Number of Rotatable Bonds) is the number of any non-ring bonds (non-cyclic bonds) bound to a non-terminally heavy (i.e., non-hydrogen) atom. C-N amide bonds are not considered due to the high rotational energy barrier, as described by Veber et al. [38]; RigidBonds (Number of Rigid Bonds) represents the number of non-flexible links, as opposed to rotary bonds (e.g., double and triple bonds, links involved in cyclic systems and amide bonds); NumCharges (Number of Charged Groups) represents the number of task groups (e.g., 1 for a compound with a carboxylic acid group); TotalCharge (Compound Total Charge) is the total number of charges of the compound; n_carbon (Number of Carbon Atoms) is the number of carbon atoms; n_hetero (Number of Heteroatoms) is the number of non-carbon atoms (not including hydrogen). The number of Heavy Atoms is the number of heavy, non-hydrogen atoms; ratioH_C (het/carbon atoms ratio) represents the ratio of the number of non-carbon atoms to the number of carbon atoms. Solubility represents the aqueous solubility (mg/L) estimated by the ESOL method [36]. Fsp3 (carbon bond saturation) is defined as the number of sp3 hybridized carbon atoms/total carbon number. This descriptor also may be correlated with the melting point and solubility [39]. StereoCenters (number of Stereocenters), the presence of stereo centers (also the number of chiral centers), is a descriptor of the complexity, which also increases with discovery through clinical studies performed on drugs. Solubility Forecast Index represents the water solubility as estimated by the method of Hill et al. [40]. TrafficLights (Oral PhysChem Score) is a widely used chemical physical oral score by Bayer, according to Lobell et al. [41].

As compliance with the rules of Lipinski, Egan, Veber, Pfizer, and/or GSK does not always lead to a “drug-like” molecule [42], FAFDrugs4.0 software (Paris Diderot University, France) introduced the drug-like filter. This filter considers that a drug-like compound has the following physico-chemical properties: 100 Da ≤ MW ≤ 600 Da, −3 ≤ logP ≤ 6, HBD ≤ 12, HBA ≤ 7, tPSA ≤ 180 Å2, Rotatable Bonds ≤ 11, Rigid Bonds ≤ 30, Rings ≤ 6, MaxSyzeSystemRing ≤ 18, 3 ≤ Number of carbon atoms ≤ 35, 1 ≤ Number of heteroatoms ≤ 15, 0.1 ≤ H/C ratio ≤ 1:1, charges ≤ 4, −4 ≤ total charge ≤ 4 [33]. FAFDrugs 4.0 also permits considering the Lilly MedChem Rules [43,44], a set of 275 rules used to identify compounds that may interfere with biological assays, allowing their removal from screening sets; the main reasons for rejection include reactivity, interference with assay measurements, protein damage, instability and lack of drug ability. Another capability that is offered by the FADrugs4.0 (Paris Diderot University, France) web server is the prediction of compounds inducing phospholipidosis, the accumulation of excessive phospholipids in tissues [45]. Within this study, we have considered the “drug-like soft” filter. We have also computed the Lilly MedChem rules (the applied demerit level being “regular”) and have predicted phospholipidosis inducers.

For simulations, the FAFDrugs4.0 tool (Paris Diderot University, France) was used based on the following rules [34]:

- The Lipinski Rule—The Rule of the 5 Violations (Ro5) (n_LipinskiViolations) The basic rule according to Lipinski et al. [46] which states that for four properties, MW ≤ 500, HBD≤ 5, HBA≤ 10 and logP ≤ 5. If two properties are outside the domain, poor absorption or permeability is possible, even acceptable (/max_lipinski).
- The Veber Rule, which may be good or not good, states (according to Veber et al. [33,38]) that the following conditions must be met: rotatable Bonds ≤ 10 and tPSA ≤ 140 Å or HBD + HBA ≤ 12
- The EGAN rule, or Bad/Good oral bioavailability rule, which also may be good or not good, stipulates, according to Egan et al. [47], that the following conditions must be met: 0 ≥ tPSA ≤ 132 if −1 ≥ logP ≤ 6
- The GSK 4/400 rule limits the logP values of the considered compounds to be less than 4 and the molecular mass (MW) to be less than 400 Da, and requires that the generated ADMET profile be favourable [43]: logP < 4 and MW < 400.
- Pfizer rule 3/75 specifies that compounds with logP > 3 and low tPSA < 75 are about 2.5 times more likely to be toxic than to be clean [44].
- State (Compound Final Status) represents the state of the compound after all the rules have been applied (i.e., accepted, intermediate or response—Accepted, Intermediate or Rejected).

TrafficLights or Oral PhysChem Score is a physico-chemical oral score widely used by Bayer HealthCare (Wuppertal, Germany) [41]. Furthermore, the DrugLikeSoft filter under FAFDrugs4.0 (Paris Diderot University, France) was considered for filtering the investigated vitamins [34].

The simulations in FAFDrugs4.0 (Paris Diderot University, France) used sdf files for each vitamin from the present simulation with the parameters presented here and the rules presented as limitations. All the simulations used the DrugLikeSoft filter under FAFDrugs4.0 (Paris Diderot University, France).

## 3. Results

### 3.1. Position of Physico-Chemical Properties of the Vitamins

**Figure 1:**
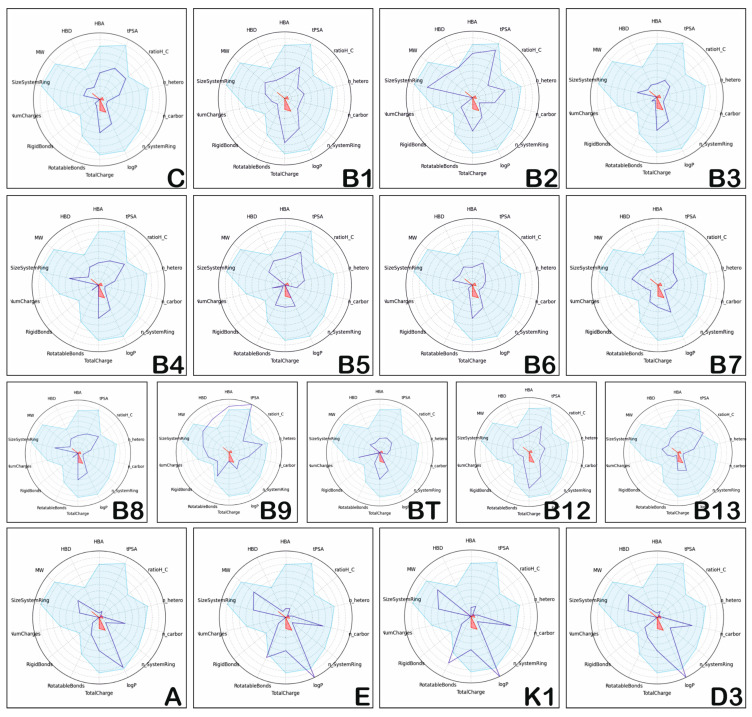
Position of physico-chemical properties of the hydrosoluble vitamins and liposoluble vitamins.

The radar graphs for the positioning of the physico-chemical properties of hydro- and liposoluble vitamins (blue dark lines) in comparison to the physico-chemical space of the drug-like compounds (light blue areas) are illustrated in Figure 1. The figure contains the following physico-chemical parameters: MW, HBD, HBA, tPSA ratioH_C, n_hetero, n_carbon, n_SystemRing, logP, Total Charge, Rotatable Bonds, Rigid Bonds, Num Charges, Size System Ring, displayed in pie chart form. These figures were obtained by using sdf files from the site of each vitamin (https://pubchem.ncbi.nlm.nih.gov/compound) and FAFDrugs 4.0 (Paris Diderot University, France) to calculate and compare the physico-chemical space of the drug-like compounds (light blue areas) and generate the radar plots.

Based on the data presented in Figure 1, the following results can be highlighted: hydrosoluble vitamins usually have their physico-chemical properties positioned in the light blue area, similar to the drug-like compounds; there is one exception, vitamin B9, that has several physico-chemical properties (HBD, HBA, and tPSA) positioned outside of the light blue area, suggesting dissimilar properties compared to drug-like compounds and low oral bioavailability. On the other hand, the liposoluble vitamins E, K1, and D3 have logP values higher than those corresponding to orally administrated drugs.

### 3.2. Complexity of Hydro-Soluble Vitamins and Liposoluble Vitamins

Structural complexity is an intuitive concept that refers to the locally defined structural features of chemical compounds, such as flexibility (ratio between rotatable and rigid bonds), fraction of sp3 (Fsp^3^) atoms, number of rigid and rotatable bonds, number of chiral centres, and rings (more precisely, Flexibility, n_System Ring, StereoCenter, Rotatable Bonds, Rigid Bonds, SizeSystemRing and Fsp^3^) [41]. These parameters can be used to qualify compounds, but there are no studies reporting on the values required for filtering and guiding the selection of compounds [48]. Consequently, structural complexity is difficult define and quantify, and is usually correlated with target selectivity of the chemical compound, as well as with its safety [49].

**Figure 2:**
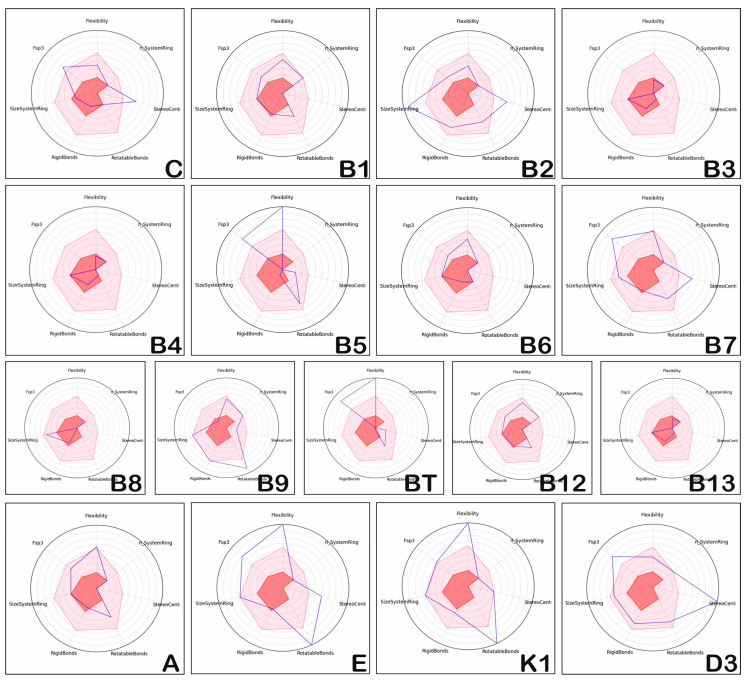
Position of complexity of hydro-soluble vitamins and liposoluble vitamins.

The positioning of the physico-chemical properties which confer structural complexity to the investigated vitamins is presented in Figure 2.

The positioning of the physico-chemical properties of hydro and liposoluble vitamins (the dark line in Figure 2) corresponds to the properties of most compounds with proven therapeutic potential (the light red area corresponds to the area of chemical compounds with biological activity and oral bioavailability). Studying Figure 2, we can state that the physico-chemical properties reflecting the structural complexity of vitamins (blue lines) do not correspond to those observed for drug-like compounds (light red areas). Vitamins C, B1, B9, B12 and A have physico-chemical properties that usually fit in the light red area, vitamins B3, B4, B6, B8 and B13 show a reduced structural complexity, and vitamins B2, B5, B7, BT, E, K1 and D3 illustrate increased structural flexibility. The same is true of the hydro-soluble vitamins, C, B2, B5, B7, B9 and BT. Moreover, vitamins C, B3, B4, B6, and B13 have properties in the forbidden area (dark red area).

Regarding vitamin E, the values of Fps3, Flexibility, StereoCenter, and RotatableBonds are higher, and RigidBonds and n_SystemRing are in the forbidden zone limit; the rest of the properties correspond to those of the majority of the therapeutically proven compounds (that is, most of the potentially therapeutic compounds have values in the light red area).

### 3.3. Assessment of the Permeability and Metabolic Stability of the Hydrosoluble and Liposoluble Vitamins

**Figure 3:**
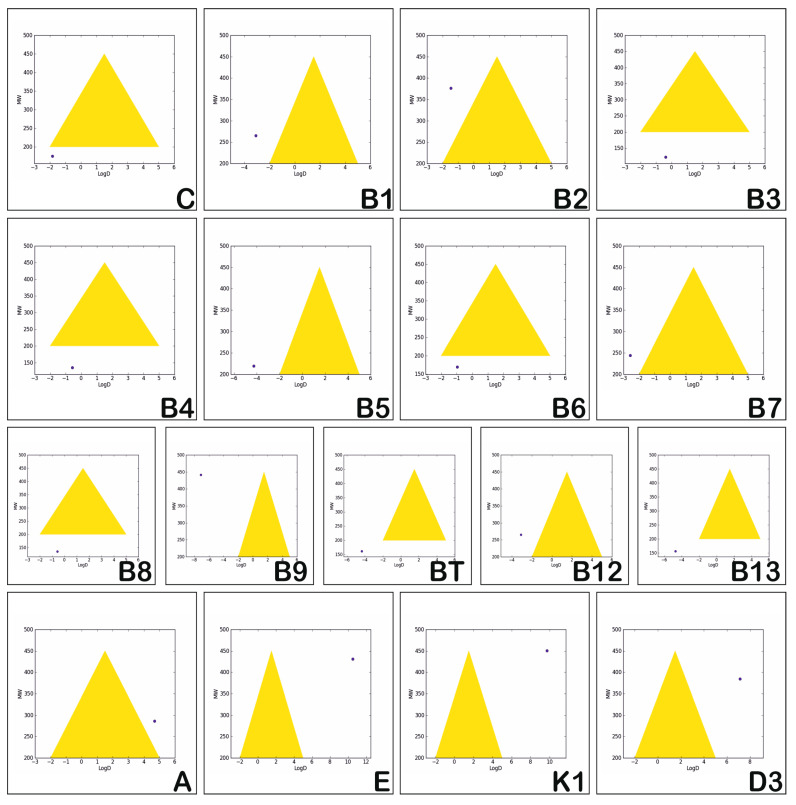
Assessment of the permeability and metabolic stability of the hydrosoluble and liposoluble vitamins.

Assessment of the in vitro permeability and metabolic stability of the investigated vitamins is based on the “Golden Triangle” visualization tool, and is illustrated in Figure 3. The properties involved are logD (the logP of compounds at physiological pH = 7.4) and MW [50]. The values of these two properties, which correspond to compounds reflecting optimal permeability and good metabolic stability, lead to a triangular shaped area named the “Golden Triangle”. Molecules having logD and MW values situated within this area are visualized by a black point and are considered as obeying the rules and reflecting good in vitro permeability and metabolic stability. Molecules having low logD and high MW values reflect low permeability and those having high values for both logD and MW reflect low metabolic stability [51]. These two situations point to a dark blue point that is situated outside the golden triangular area.

Figure 3 illustrates that none of the investigated vitamins reflects permeability and metabolic stability. The liposoluble vitamins have high values for both logD and MW, which reflects poor metabolic stability, compared with the hydrosoluble vitamins which usually have low values for both logD and MW, reflecting low permeability.

### 3.4. Statistical Analysis of Positioning of the Properties of the Hydro- and Liposoluble Vitamins (Red Point) against Properties of Other Drugs Found in DrugBank (Yellow Dots) and Edrugs (Blue)

**Figure 4:**
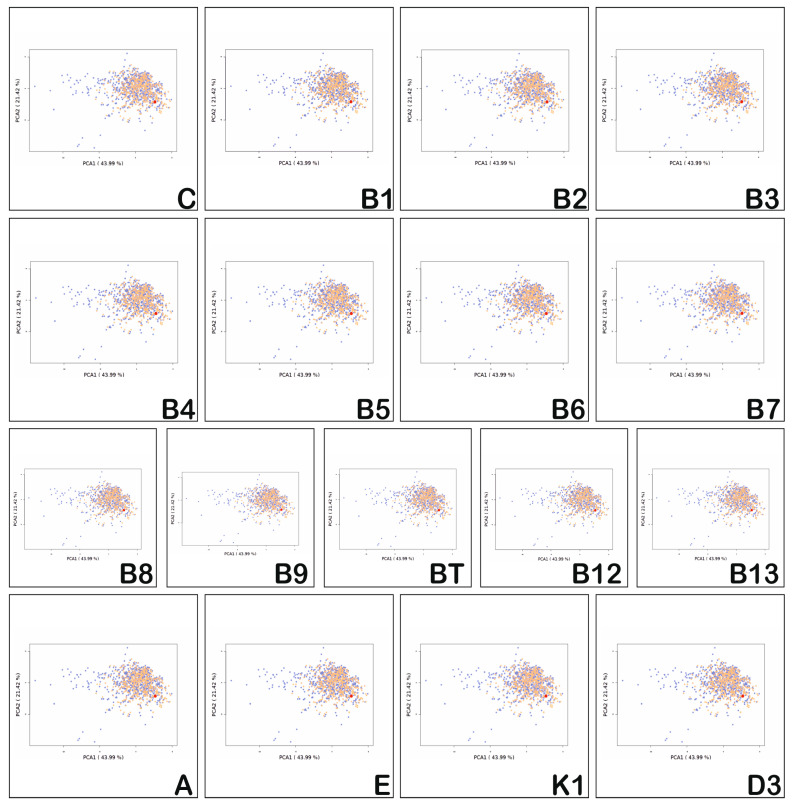
Statistical analysis of the positioning of the properties of the hydro- and liposoluble vitamins (red point) against the properties of other drugs found in DrugBank (orange dots) and Edrugs (blue).

Figure 4 shows the application of the Principal Component Analysis (PCA) method of 15 physico-chemical descriptors to the investigated vitamins, as well as the positioning of the vitamin (red dot) against the chemical space of the 466 orally bioavailable compounds extracted from DrugBank [52] (orange dots) and the 916 compounds extracted from EDrugs [53] (blue dots). All of the hydro- and liposoluble vitamins have properties reflecting low oral bioavailability.

### 3.5. Estimation of Oral Absorption of the Hydrosoluble Vitamins and Liposoluble Vitamins

**Figure 5:**
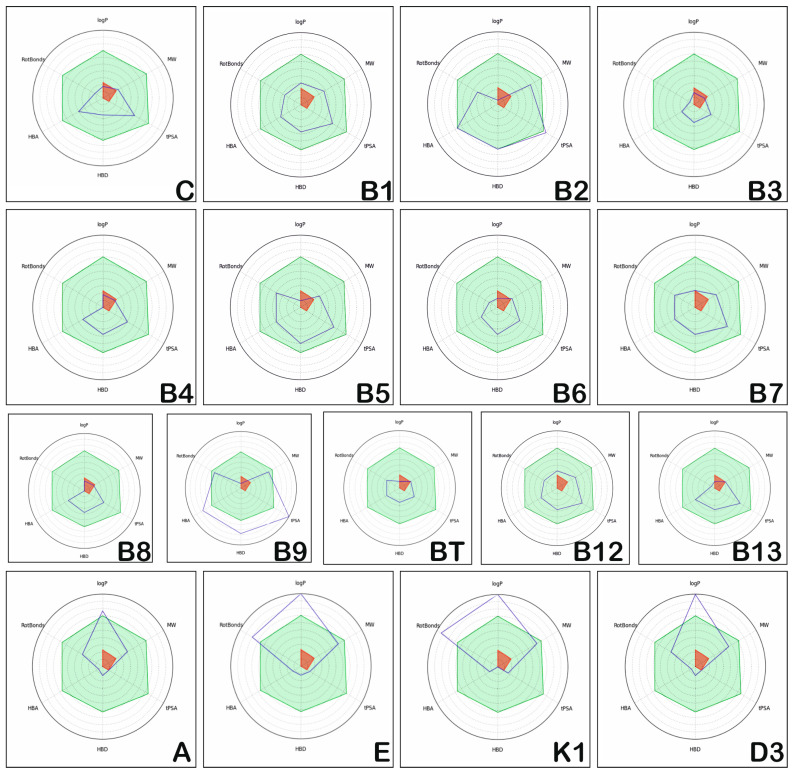
Estimation of oral absorption of the hydrosoluble and liposoluble vitamins.

The radar plots emphasising the oral absorption estimations of the investigated vitamins are illustrated in Figure 5. The physico-chemical properties considered for assessing the oral absorption are logP, rotatable bonds, HBA, HBD, tPSA, and MW. The optimal range for these descriptors is represented by the light green region.

Figure 5 illustrates that vitamins C, B1, B2, B3, B4, B5, B6, B7, B12, BT, B13, and A have almost all of their physico-chemical properties situated in the optimal regions, and as such reflect good oral absorption. Vitamins B9, K1, E, and D3 have numerous properties outside of the optimal area and reflect low oral absorption. Vitamins A and D3 have logP higher than normal, and vit E and K1 have both logP and RotBonds higher than normal, while Vitamin B9 has HBA, HBD, tPSA much higher than normal.

On the contrary, vitamins C, B2, B5, B6, B3, B4, B8, BT and B13 have values in the restricted area, with logP much smaller than normal. Vitamin BT and B13 are at the limit in terms of MW, while vitamin B3 has a lower MW and B4 and B8 have logP, RotBonds, and MW lower than normal, situated in the restricted area.

### 3.6. Toxicity Assessment of the Hydrosoluble and Liposoluble Vitamins

**Figure 6:**
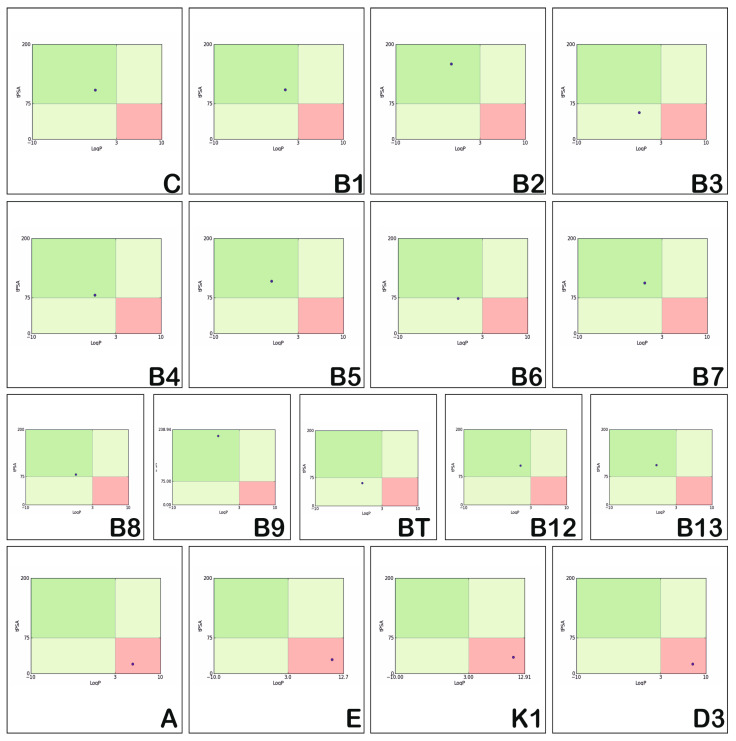
Toxicity assessment of the hydrosoluble and liposoluble vitamins.

The use of the Pfizer’s rule for evaluating the toxicity of the investigated vitamins is illustrated in Figure 6. Pfizer’s rule considers logP and tPSA as molecular descriptors. According to this rule, the chemical space is divided into four regions: a dark green region for logP < 3 and tPSA > 75 Å^2^, corresponding to compounds without toxicity, and a light green region for logP < 3 and tPSA < 75 Å^2^, corresponding to Pihan et al. [53].

Figure 6 reveals that the physico-chemical properties of the hydrosoluble vitamins (blue dots) correspond to dark green regions (the left-upper corner region) and they are either not toxic (C, B1, B2, B4, B5, B7, B8, B9, B12, and B13) or they reflect low toxicity (B3, B6, and BT) and correspond to light green regions. On the other hand, the properties of the liposoluble vitamins (A, E, K1, and D3) are located in the light red area (the right-lower corner region) and reflect a relative toxicity.

On the contrary, vitamins B3 and BT show reduced toxicity. Moreover, vitamins C, B1, B2, B5, B7, B9, B12 and B13 show no toxicity. However, vitamins B4, B6, and B8 show no toxicity, but are very close to the area of reduced toxicity.

In terms of toxicity, Vitamins B1, B2, B7 and B9 have been shown to present toxicity even if they normally do not show toxicity, because they do not respect some of the Lilly–MedChem rules (see Table 2). Vitamin B3 is in the region without toxicity, vitamin B6 is at the edge of non-toxic and low toxicity region, and BT is in the low toxicity region. Pfizer’s BT rules also indicate a tPSA = 60.360 < 75, which places it in the region with low toxicity; thus, we cannot say it has no toxicity, just as for BT (tPSA = 55.980 < 75) and (B6: tPSA = 73.58 < 75).

Table 2 shows that liposoluble vitamins generally have high bioavailability. It can be observed that for all liposoluble vitamins, Lipinski RO5 rule presents one violation for each of them: for vitamin A, logP = 5.68 > 5, for vitamin D3 logP = 5.95 > 5, for vitamin E logP =10.7, and for vitamin K1 logP = 10.91 > 5. In addition, all of the liposoluble vitamins present violations of the Pfizer 3/75 rule with logP > 3, since these values are already higher than 5. tPSA (topological Polar Surface Area) has to be >75; however, for vitamin A, tPSA = 20.230, for vitamin D3 tPSA = 20.230, for vitamin E tPSA = 29.460, and for vitamin K1 tPSA = 34.14. Thus, for all these reasons, liposoluble vitamins fall in the region with toxicity. Vitamin K1 has one violation of Lilly–MedChem rules.

On the other hand, Table 2 gives us information regarding the oral bioavailability profile and toxicity profile for the hydro- and liposoluble vitamins. It can be seen that vitamins B8 and B9 violate Lipinski’s rule:

- for vit B8, HBA = 12 > 10 and HBD = 6 > 5
- for vit B9, HBA = 13 > 10 and HBD = 7 > 5

Vitamins B1, B2, B7, and B9 present a relative toxicity because they violate the Lilly–MedChem rules. Vitamin B3 is in the non-toxic region, B6 is in the non-toxic region and the low toxicity region, and BT is in the low toxicity region. This assertion is also indicated by the fact that these vitamins violate the Pfizer rule due to the fact that the area of the topological surface, tPSA, has values not allowed by the Pfizer rule: for BT, tPSA = 60.360 < 75, which places it in the low toxicity region; in addition, for B3 tPSA = 55.98 < 75, and for B6 tPSA = 73.58 < 75.

Table 2 reveals that fat-soluble vitamins in general do not have high bioavailability. It can be seen that all fat-soluble vitamins violate Lipinski’s RO5 rule, as follows: for vitamin A, logP = 5.68 > 5, for vitamin D3 logP = 5.95 > 5, for vitamin E logP = 10.7, and for vitamin K1 logP = 10.91 > 5. From the point of view of the toxicity it has been revealed that the fat-soluble vitamins violate the Pfizer 3/75 rule that requires logP > 3, and for all fat-soluble vitamins these values are > 5; in addition, the tPSA (polar topological area) must be >75; however, their values are as follows: for vitamin A, tPSA = 20.23, for vitamin D3 tPSA= 20.23, for vitamin E tPSA = 29.46, and for vitamin K1 tPSA = 34.14, which leads to all fat-soluble vitamins being in the toxicity region. Table 2 also shows that only vitamin K1 violates the Lilly–MedChem-rules.

Predictions show that water-soluble vitamins generally have high bioavailability (except B8 and B9, which do not meet the Lipinsky rule). They also have no toxicity, except for vitamins B3, B5 and BT, which have a low toxic potential, and B6, which is at the limit of the reduced toxicity zone. None of the investigated vitamins is predicted to produce phospholipidosis.

## 4. Discussion

The consumption of vitamin and mineral-based supplements as well as their use in fortifying foods have drastically increased in recent decades due to a higher rate of deficiencies reported in industrialized countries, despite lack of knowledge about their complete safety profile and their real benefits. Understanding the exact and complete role of the vitamins, their safety profiles, and their use is crucial. Not only can liposoluble vitamins be toxic, but hydrosoluble vitamins may also exert some toxic effects, or may simply not be absorbed by the body if there is no deficiency. Moreover, the optimal intake of each vitamin is controversial, as the current analytical techniques cannot target tissue storage, and they are not entirely fitting for all of the intended goals of therapy [14,54,55,56].

The present paper describes the toxicity profiles of several vitamins based on the FAFDrugs 4.0 (Paris Diderot University, France) internal rules. Table 1 presents the chemical structures involved in toxicity that could be detected by FAFDrugs 4.0. (Paris Diderot University, France). Moreover, some substructures are hepatotoxic, an effect that could be found with some vitamins, as explained in the following discussion; however, some online tools are able to manage these predictions of hepatotoxicity.

The present study discusses the ADMET profiles of several hydro- and liposoluble vitamins with the following major findings: vitamin B3 and B13 have reduced complexity, and could exert some toxicity, as Figure 2 shows, compared with the liposoluble vitamins which have a higher complexity, much greater that most potentially therapeutically-proven compounds.

Indeed vitamin B3, as reported in the literature, is used mainly in the treatment of atherosclerotic diseases and dyslipidaemia, being an extremely effective lipid regulating drug, although with some problematic side effects such as flushing and hepatotoxicity [57].

Vitamin B13 was found to have an important neuroprotective role, with several side effects that were observed in animal models such as severe liver steatosis, hepatomegaly, and tumor development [58].

Vitamin E was found to have the greatest complexity of all the studied vitamins (followed by vitamin D), and by correlating this information with the actual literature vitamin E was reported to be involved in several important processes in the human body: maintaining the normal morphology of erythrocytes, inducing anti-platelet, anti-inflammatory, and anti-proliferative effects, and thereby having a protective role against several pathologies including cardiovascular, neurological and immunological diseases, arthritis, and cancer [59].

In the present study, all of the analysed vitamins were found to have low permeability and poor metabolic stability, as Figure 3 shows. Correlating this information with the present literature data and with previously reported in vivo studies, we can state that the pharmacokinetics of vitamins are very complex and closely influenced by their individual pharmacokinetics, making it very difficult to be study their performance and interrelation; this field requires further studies to be completely elucidated [2,55].

As was expected and already known, fat soluble vitamins were found to have the best oral absorption and the best bioavailability, as was as vitamin B9 [1,2]. Vitamin B9 is the synthetic form of the “natural” dietary folate (L-5-methyl-tetrahydrofolate) found in fortified foods, supplements, and pharmaceuticals. Having no coenzyme activity, it must be reduced within the cell to its metabolically active form, tetrahydrofolate; the advantage of this synthetic form is its greater stability compared with the natural folates. Moreover, vitamin B9 is absorbed directly in the small intestine, whereas dietary folates require hydrolysis prior to absorption [14,56,57,58,59,60].

Vitamin B1 and B7 were found to have good oral absorption and good bioavailability. In our study, vitamin B3 was found to have decreased oral absorption, although bolus doses up to 3–4 g of nicotinic acid were reported in the literature to have good absorption [10].

Regarding their toxicity, as reported in the literature and already mentioned, we found that the liposoluble vitamins have the greatest risk of bioaccumulation (i.e., the highest toxicity) compared with hydrosoluble ones, although of these, vitamin B1, B2, B7 and B9 could have a higher risk of accumulation. At the moment, the present data regarding thiamine and riboflavin toxicity are inconsistent and the studies performed show a high risk of bias, as reported by several reviews [61,62,63].

The increased toxicity of liposoluble vitamins is due to their pharmacokinetic properties within the human body, which are directly correlated with their physico-chemical properties. In brief, they are absorbed by the micelles of the small intestine under the action of bile and pancreatic secretion. Being stored by the chylomicrons, they travel into the lymphatic system, then enter into the blood stream and are released into the tissues for use or deposits under lipoprotein lipase action. Their risk of accumulation is due to their storage in tissue and lower degree of elimination [1]. Due to their risk of accumulation, with constant consumption over time they might elicit toxicity. However, the phenomenon can be simply eliminated by arresting the consumption of pharmaceutical supplements and/or fortified foods. On the other hand, in the case of water-soluble vitamins the excess will be eliminated by the human body and not stored as in the case of the liposoluble vitamins [1]. However, closer monitoring of hydrosoluble vitamin side effects should still be carried out in order to have an exact safety profile.

Although it is widely accepted and known that vitamins are vital substances for the proper functioning of the human body (important because they cannot be synthesized within the body), there is still a substantial need for information, as the present literature is insufficient and sometimes contradictory. More information and further research are needed regarding their pharmacological properties such as absorption (exact form), adequate dose, complete action and function in the body, risk of accumulation, and toxicity. 

Regarding the effects of routine supplementation, it is highly needed in patients with nutritional deficiencies; however, caution should be taken for those without nutritional depletion as vitamins can induce toxic effects, nutritional deficiencies being most problematic under clinical conditions (vulnerable patients, pediatric, institutionalized, or elderly patients, and those with other associated pathologies).

Particular attention should be paid when consuming vitamins, as the World Health Organization (WHO) has recently reported two different counterfeit vitamin A capsules in the Chad region of Africa. Both items were underdosed, containing a percentage of 68.6% and 64.4% of the manufacturer’s declared concentration [64]. Since this unfortunate event has occurred, there may exist the possibility of overdosed items that contain vitamins which can accumulate into the body and cause severe side effects; therefore, vitamin intake should not be considered completely inoffensive. Regarding this aspect, according to currently available information, the main factor leading to vitamin D toxicity is considered to be manufacturing failure which implicitly leads to labeling errors. Several cases of Vitamin D intoxication were reported due to overdosed formulations (fish oil or oral formulas) that contained between 1000–4000 times more vitamin D than the stated content on the label [65]. In addition, fortified foods with high amount of vitamin D have induced hypervitaminosis D or intoxication [66]; thus, these types of products are governed by strict regulations in the United Kingdom [67]. Vitamin A has also been responsible for inducing severe toxicity; in addition, its association with alcohol leads to a synergistic hepatotoxic activity that can lead to liver failure [68]. Also, the use of vitamin E acetate as a vehicle for diluting either tetrahydrocannabinol (THC) oil or nicotine-based e-liquids for vaping products has recently been associated with bronchoalveolar lavage in patients suffering e-cigarette/vaping-associated lung damage, which is an important concern taking into account that these types of devices are developing rapidly; so far over 2800 people have suffered e-cigarette/vaping-associated lung injury in the United States (US) alone [69]. Besides the aforementioned liposoluble vitamins, the hydrosoluble ones such us vitamin C may induce side effects if not administrated properly; for example, people suffering from glucose-6-phosphate deficiency treated with high concentration of vitamin C may suffer of hemolysis, this effect being amplified in patients with paroxysmal nocturnal hemoglobinuria. In addition, administration of oral doses higher than 1000 mg of vitamin C leads to a 41% increase in the rate of urinary stone formation [70]. New data has also revealed that high concentrations of vitamin C affect neural stem progenitor cells by inducing apoptosis through impaired production of GSH and NAD+ [71].Vitamin B6 in pyridoxine form has been associated with neuropathic symptoms in patients treated with concentrations not exceeding the daily dose of 0.025 g recommended by the European Food Safety Authority [72], and vitamin B12 in cyancobalamin form has been found to induce toxicity when administrated in multiple high doses in the case of a 24-year old woman suffering from two different autoimmune diseases (pernicious anemia and thyroiditis) [73]. Nevertheless, as recently reported by Olson et al. [74], more than 60,000 cases of vitamin-related toxicity are registered in the US each year.

As a response to the side effects observed in vitamin administration, a plethora of free online tools give the opportunity to test side-effects, absorption rate and other pharmacokinetic features of vitamins, such us ADMETlab is one. Performing analyses with web portals such as FAFDrugs 4.0 (Paris Diderot University, France) may constitute an interesting approach for future studies.

However, this study demonstrated that apart from the well-known toxicity of fat-soluble vitamins, some of the water-soluble ones could also exert toxicity, and thus routine supplementation with vitamins or ingestion of fortified foods should not be considered harmless or, on the contrary, to have great benefits.

At the moment, much more research is needed for a clear understanding of the action and importance of vitamins in the human body in order to ensure optimal and safe treatment supplementation; thus, a varied diet based on fruits and vegetables should always be considered the first option for assuring sufficient intake of these vital substances. In addition, caution and follow-up investigation are required in patients taking vitamin supplements for long periods of time without any medical or pharmaceutical advice through self-medication, as they can induce more side effects than benefits.

The present theoretical study requires further in vivo confirmation, as many physiological and physio-pathological factors can interfere with the action of these vitamins in the human body.

The results of this research indicate that fat-soluble vitamins in general do not have high bioavailability. It can be seen that all fat-soluble vitamins violate Lipinski’s RO5 rule. From the point of view of toxicity, it was found that the fat-soluble vitamins violate the Pfizer 3/75 rule that imposes logP > 3. Predictions show that water-soluble vitamins generally have high bioavailability (except B8 and B9, which do not meet the Lipinsky rule). They also have no toxicity, except for vitamins B3, B5, and BT, which have low toxic potential, and B6, which is at the edge of the reduced toxicity zone.

Liposoluble vitamins have no oral bioavailability and have higher toxic potential. None of the fat-soluble vitamins comply with the Pfizer rule (as expected). Liposoluble vitamins could have adverse effects related to their physico-chemical properties, such as too high a logP in vitamins E and D3.

A modification of the structure of vitamins A, D3, E, and K1 may be useful to effect modification of their physico-chemical properties so as to avoid causing adverse effects.

Summing up, the vitamins presented in this study are compounds accepted as having therapeutic properties with good oral bioavailability; however, some of them, including all the liposoluble vitamins, reflect toxicity. Moreover, vitamins B3 and BT showed a reduced toxicity. It can be said that vitamins B8 and B9 violate two of Lipinski’s requirements. In terms of toxicity, Vitamins B1, B2, B7 and B9 have been shown to present toxicity even if they normally do not show this toxicity, because they do not respect some of the Lilly–MedChem rules. Vitamin B3 is in the region without toxicity, vitamin B6 is at the limit of the non-toxic and low toxicity region, and BT is in the low toxicity region. This also indicates that BT violates Pfizer’s rules. For all liposoluble vitamins, the Lipinski RO5 rule presents a single violation for each of them. From the point of view of toxicity, we can observe that all of the liposoluble vitamins present violations of the Pfizer 3/75 rule. For all these reasons, liposoluble vitamins are in the region with toxicity. Vitamin K has one violation of Lilly–MedChem rules.

The limitations of the current study using the present method are related to its rudimentary explanations of several characteristics of the analysed compounds, such as (i) lipophilicity (information on terms such as iLOGP, XLOGP3, WLOGP, MLOGP, etc.), (ii) pharmacokinetics (information related to gastrointestinal absorption, penetration of the blood–brain barrier, the impact of certain izoenzymes of cytochrome P450 in the metabolic process), and (iii) medical chemistry of the molecules studied.

## 5. Conclusions

Computational studies of the ADMET type, such as the one presented here, provide information on the oral bioavailability profile and toxicity profile, and are useful before introducing a new drug on the market to see if its properties are appropriate.

An analysis of the information from the specialized literature on these vitamins and their effects is required in the future. These results should be compared with data known from the literature on the biological effects of these vitamins, and discussed based on what is known and what has been predicted.

The current study is of great importance, as it presents information about the vitamins investigated herein as compounds accepted as having therapeutic properties and good oral bioavailability. However, some of them, including all the liposoluble vitamins, reflect relative toxicity. In addition, hydrosoluble vitamins such as vitamins B1, B2, B3, B7, B9, B12 and BT show a reduced toxicity, while vitamin B6 is at the edge of the non-toxic and low toxicity region.

In addition, this study highlights the concerns regarding the relationship between biological toxicity correlated with the physico-chemical properties of hydro- and lipo-soluble vitamins, along with newly discovered reports on vitamin toxicity.

## Figures and Tables

**Table 1:** 2D structural formulas and the main biological functions for hydro- and lipo-soluble vitamins.

Hydro-Soluble Vitamins	
Vitamin C—Ascorbic acid—C_6_H_8_O_6_, MW = 176.12 g/mol. [3]	Vitamin B1—Thiamine—C_12_H_17_N_4_OS^+^, Molar mass MW = 265.35 g/mol.
- cofactor for the biochemical reactions having as a substrate monooxygenases, dioxygenases and oxygenases - important role in the synthesis of collagen, carnitine and catecholamines, conversion of cholesterol to bile acids and inducing of antioxidant effect (it scavenges oxygen and nitrogen species) [3]	- functions as a precursor for TDP (thiamin diphosphate), which is a co-enzyme for the enzymes involved in carbohydrate and branched chain AA (amino acid) metabolism and energy-yielding reactions [3]
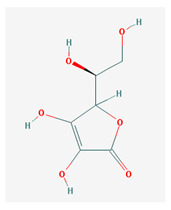	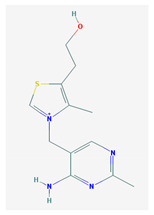
Vitamin B_2_—Riboflavin—C_17_H_20_N_4_O_6_, Molar mass MW = 376.36 g/mol.	Vitamin B_3_—Niacin—C_6_NH_5_O_2_, Molar mass MW = 122.13 g/mol.
- functions as a coenzyme in several reactions of oxidation and reduction in metabolic processes and energy production (via respiratory chain) within the body - involved in the metabolism of niacin, vitamin B6, iron, folate cycle and homocysteine [3]	- Vitamin B_3_ or niacin (the generic term) consists of nicotinic acid and nicotin amide. It can be synthesized in the human body from tryptophan; however, low plasma levels of iron, riboflavin or piridoxin will decrease the conversion of tryptophan to niacin [3] - It functions as a co-enzyme for the transfer of hydrogen, together with numerous dehydrogenases. Nicotinamide is required for lipid metabolism, tissue respiration and glycogenolysis [4]
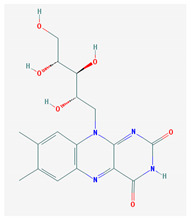	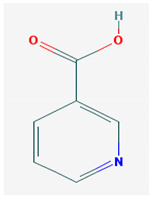
Vitamin B_4_—Adenine—C_5_H_5_N_5_ molar mass MW = 135.13 g/mol.	Vitamin B_5_—Pantothenic acid—C_9_H_17_NO_5_, molar mass MW = 219.24 g/mol
- Vitamin B4 is no longer considered a true vitamin. It functions mainly to enhance the energy producing processes (together with vitamin B2 and B3) in the human body. - important key for DNA and RNA synthesis, protein synthesis, cell and tissue development, immune system [5,6]	- is a ubiquitous vitamin (deficiency being extremely rare), component of coenzyme A (CoA) and acyl carrier group proteins; involved in fatty acid metabolism [3]
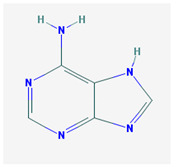	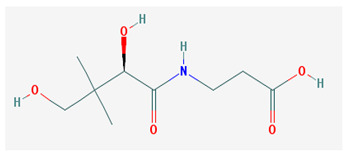
Vitamin B_6_—Pyridoxine—C_8_H_11_NO_3_, MW = 169.18 g/mol, Pyridoxamine C_8_H_12_N_2_O_2_, MW = 168.2g/mol, pyridoxal—C_8_H_9_NO_3_, MW = 167.16 g/mol.	Vitamin B7—Biotine—C_10_H_16_N_2_O_3_S, MW = 244.31 g/mol.
- functions as an essential co-enzyme for amino acid metabolism, glycogen and sphingoid bases [4]	- This water-soluble vitamin acts as a co-enzyme for several carboxylases implicated in the synthesis of fatty acids, gluconeogenesis and catabolism of branched-chain amino acids [3]. Animal studies show that it is essential for normal fetal development [7]
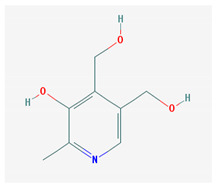	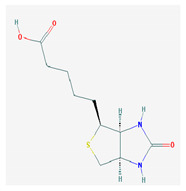
Vitamin B8—Adenosine monofosfat, adenilic acid-C_10_H_14_N_5_O_7_P, MW = 347.22 g/mol.	Vitamin B9—folic acid —C_19_H_19_N_7_O_6_, MW = 441.40 g/mol.
- Is no longer considered a true vitamin, but rather a nucleotide found in RNA. - used as a dietary supplement either to stimulate immune system activity or as a sweetener in a low-calorie diet [8]	- functions as a co-enzyme for the enzymes implicated in one-carbon metabolism; important for the DNA and RNA synthesis. - of great significance for the methionine cycle which will is a precursor in the synthesis of DNA, hormones, neurotransmitters, membrane phospholipids and proteins [3] - necessary for erythropoiesis, synthesis of purine and thymidylate, metabolism of amino acids (glycine, methionine and histidine) - can offer protection against cardiovascular pathologies and some forms of cancer [9]
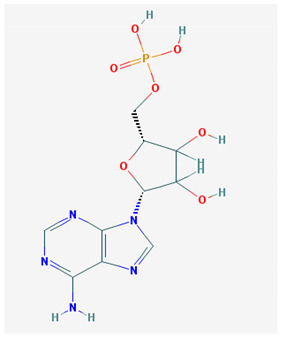	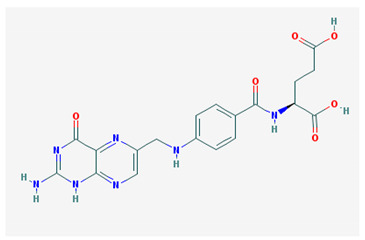
Vitamin BT—L-carnitine or Levocarnitine—C_7_H_15_NO_3_, MW = 161.199 g/mol.	Vitamin B13—Acid orotic—C_5_H_4_N_2_O_4,_ MW = 156.10 g/mol.
- an enantiomer with an important role in energy production at the cellular level - an essential nutrient in the metabolism of fats and a peripheral antagonist of thyroid hormone action in some tissues [10]	- has an important neuroprotective role by exhaustion of pyrimidines. The production of the stump itself is necessary for the development of the body. - plays a role in regulating transcription of crucial genes; the synthesis of the UMP enzyme may also have the role in transient cerebral ischemia, ischemic disorders, and frontal cerebral ischemia [11] in the nucleus
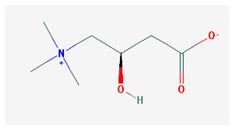	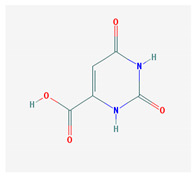
Vitamin B12—Cyanocobalamin, hydroxocobalamin, methylcobalamin, adenosylcobalamin— C_6_3H_88_CoN_14_O_14_P, MW = 1355.388 g/mol	
- Main source of vitamin B12 is the intake of animal proteins (meat, fish, milk products, egg yolk) - Involved in DNA synthesis (cofactor), amino acid and fatty acid metabolism, myelin synthesis and red blood cell maturation in bone marrow - Deficiencies may appear mostly due to impaired absorption (lack of gastric intrinsic factor) or achlorhydria [12,13]	
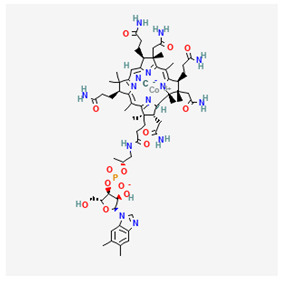	
Lipo-soluble vitamins	
Vitamin A—Retinol—C_20_H_30_O, MW = 286.45 g/mol, Retinal—C_20_H_28_O, MW = 284.44 g/mol, retinoic acid—C_20_H_28_O_2_, MW = 233.43 g/mol, Carotenoide; beta carotene.	Vitamin E α-tocopherol—C_29_H_50_O_2,_ MW = 430.71 g/mol, β-tocoferol—C_28_H_48_O_2,_ MW = 416.68 g/mol, γ-tocoferol—C_28_H_48_O_2,_ MW = 416.68 g/mol, δ-tocoferol—C_27_H_46_O_2,_ MW =402.65 g/mol
- an essential substance that the human body requires in small amounts for several processes, such as proper functioning of the visual system, reproduction, growth and development, immune system, cell structure, growth, differentiation, apoptosis and regeneration. It also regulates the metabolism of lipids, proteins and carbohydrates [4,14]	- It protects several substances from oxidation by free radicals, having anti-ageing properties [4] - involved in maintaining the normal morphology of erythrocytes; has anti-platelet, anti-inflammatory, and anti-proliferative properties and can induce a protective role for several pathologies such as cardiovascular, neurological and immunological diseases [14]
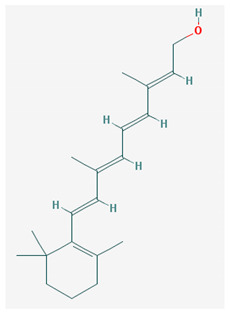	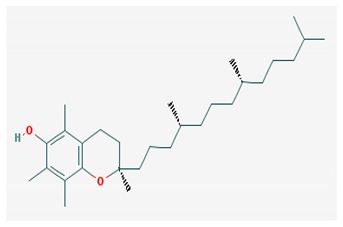
Vitamin K—includes 2 natural vitamins: vitamin K1 —phylloquinone —C_31_H_46_O_2_, MW = 450.7 g/mol and vitamin K2 —menaquinone-4 — C_31_H_40_O_2_, MW= 444.6 g/mol	Vitamin D—Ergocalciferol (D2)—C_28_H_44_O, MW = 396.65 g/mol and cholecalciferol (D3)—C_27_H_44_O, MW = 348.648 g/mol.
- Plays a key role in the coagulation process; deficiency can induce bleeding problems, while when taken orally, natural sources of vitamin K1 seem without major side effects, although increased coagulation was observed in patients taking warfarin [14]	- Vitamin D can either be synthesized from a cholesterol precursor in the skin (vitamin D3) after sunlight exposure (considered a prohormone) or it can be taken from dietary sources (vitamin D3 or D2); - has an important role in maintaining the balance of calcium and phosphate circulating levels within the body, contributing to bone mineralization, muscle contraction and other functions [4]
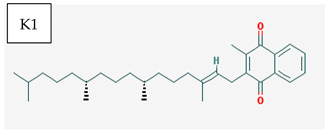 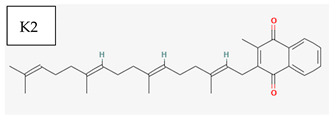	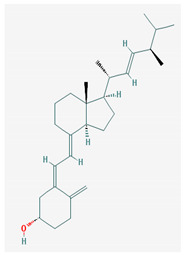

**Table 2:** ADMET profiles for hydro- and liposoluble vitamins.

Vitamin	Oral Bioavailability				Drug Safety Profiling Toxicity			
	Lipinski RO5	Veber Rule	Egan Rule	Bayer Oral Physchem Score	GSK 4/400 Rule	Pfizer 3/75 Rule	Phospho	Lilly Medchem Rules
							Lipidosis	
							Non Inducer	
B1	0	good	good	0	good	good	Non inducer	Quaternary aryl
B2	0	good	good	2	good	good	Non inducer	Anthracene het
B3	0	good	good	0	good	warning	Non inducer	pass
B4	0	good	good	0	good	good	Non inducer	pass
B5	0	good	good	0	good	good	Non inducer	pass
B6	0	good	good	0	good	warning	Non inducer	pass
B7	0	good	good	0	good	good	Non inducer	Biotin
B8	2	good	good	2	good	good	Non inducer	pass
B9	2	good	good	4	good	good	Non inducer	Too many atoms negative aniline hewd
B12	0	good	good	0	good	good	Non inducer	pass
B13	0	good	good	0	good	good	Non inducer	pass
BT	0	good	good	0	good	warning	Non inducer	pass
C	0	good	good	0	good	good	Non inducer	pass
A	1	good	good	2	good	bad	Non inducer	pass
E	1	good	good	5	bad	bad	Non inducer	pass
D3	1	good	good	2	good	bad	Non inducer	pass
K1	1	good	good	5	bad	bad	Non inducer	Quinone para

Each color indicates a specific feature: (i) green colour-no caution should be taken; (ii) orange–warning; (iii) red-caution.
